# Supine Percutaneous Nephrolithotomy: Preliminary experience in a single hospital

**DOI:** 10.12669/pjms.40.8.9188

**Published:** 2024-09

**Authors:** Tanweer Ahmed Naweed Bhatty, Eemaz Nathaniel, Sadaf Noureen, Nadeem Iqbal

**Affiliations:** 1 Tanweer Ahmed Naweed Bhatty Pakistan Kidney and Liver Institute, Lahore, Pakistan; 2 Eemaz Nathaniel Ibrahim Khalil, Research Officer, Department of Medical Research, Rehman Medical College, Peshawar, Pakistan; 3 Sadaf Noureen, The Groves Medical Centre New Malden, Clarence Ave, New Malden, United Kingdom; 4 Nadeem Iqbal Pakistan Kidney and Liver Institute, Lahore, Pakistan

**Keywords:** Percutaneous nephrolithotomy (PCNL), Supine position, Kidney stones, Endourology, Clavien-Dindo classification

## Abstract

**Background and Objective:**

Percutaneous Nephrolithotomy (PCNL) is recommended for large Kidney Stones. It is mostly done in prone position. However, PCNL in Supine position is another safe option. Only few centers in country are doing it and so it is challenging task to adopt supine PCNL approach in an institution initially. In this study our purpose was to assess initial experience of Supine PCNL in our center.

**Methods:**

It is a preliminary retrospective study of our first fifty-one supine PCNL procedures, performed by a single Surgeon, over Twelve months period, from April 2021 to April 2022. We managed a retrospective review of patients’ records. Analysis was completed by utilizing SPSS version 20. Implementation of Mean along with standard deviation values was utilized for continuous variables. While frequency/percentages represented categorical factors.

**Results:**

Patients mean age was 39 years, comprising of 62.74% male and 37.25% female patients. Thirty patients had their stones treated on the left side. Mean Stone burden was 3.2 cm. Most of the stones were GUYs score one and two (complexity wise). The mean procedure time 147minutes. Mean hospital stay of 2.17 days was observed in this study. Forty patients were stone free. Only seven patients (14%) had level I-II complications (Clavien-Dindo classification).

**Conclusion:**

Supine PCNL can be adopted safely in an institute if careful selection of patients is done before surgery. In our center it had acceptable success rates and few complications.

## INTRODUCTION

Percutaneous Nephrolithotomy (PCNL) is the most recommended treatment for large Kidney Stones and is considered as the “Gold Standard”.^1^ It is mostly done in prone position. First percutaneous renal puncture was performed in 1954 by a radiologist, in a hydronephrotic kidney to do antegrade pyelogram.^2^ PCNL in prone position was first reported in 1976.^3^ Initially it was done in the prone position, because of the urologists’ fear of inadvertent injury to the colon during tract formation.^4^,^5^ However, with availability of better imaging, the delineation of interposed organs between the skin and the kidney became much easier.^2^ As CT (Computed tomography) became more commonly available for preoperative assessment, it became evident that the chances of retrorenal colon is lower in the supine than in the prone position. PCNL in supine position was first reported in 1987-1988 by Valdivia.^4^ Supine PCNL showed obvious advantages with regards to ergonomics and anesthesiologic management in addition to comparable complications and success rates. Supine PCNL was later adopted as a safe treatment option after Galdakao modified technique in 2007.^5^ The supine position definitely provides some edge over prone PCNL when it comes to anesthesiological management, because of better cardiovascular and airway control, especially in emergency scenarios.^5^-^7^ In addition to these, there are less chances of injury to back bone, and a lower risk of thromboembolism by reason of the lack of inferior vena cava compression.^8^-^9^ In this study we present a single Hospital’s early experience for adopting supine position PCNL in its first 51 patients.

## METHODS

It is a preliminary retrospective study of our first fifty-one supine PCNL procedures, performed by a single Surgeon, over 12 months period, from April 2021 to April 2022. We managed a retrospective r.

This study encompassed all patients aged ≥18 years to 70 years at our hospital who received surgical treatment for renal stones by PCNL (Percutaneous Nephrolithotomy). The study purpose was to assess initial outcomes of performing supine PCNL at our department. These outcomes were assessed in terms of operative time, hospital stay, stone clearance, and post-operative complications (e.g., vomit, fever sepsis), need for blood transfusion, perinephric collection and extra need for administering pain killers.

### Ethical Approval:

The study was approved by our institution’s ethical committee (IRB No. PKLI-IRB/AP/83; dated October 14, 2022).

### Inclusion & Exclusion Criteria:

Inclusion criteria in the study comprised of renal stone size exceeding 2cm and those who went through supine PCNL. Patients who had shown bacterial growth in urine were treated according to the culture sensitivity before undergoing the procedure. However, those who had compromised renal function, previous sessions of shock wave lithotripsy, previous open stone surgery on the ipsilateral kidney, those who did not agree to undergo supine PCNL or patients who had bleeding disorders were excluded from the study outcomes.

Prior to the supine PCNL, patients were counselled regarding the procedure and the possible outcomes and an Informed consent was then taken. They underwent essential preoperative assessment including complete hematological analysis, renal function tests, KUB radiograph, serum biochemistry, coagulation profiles, urine cultures. Stone’s location and size were assessed by non-contrast computed tomography (CT). Stone complexity was estimated by utilization of Guy’s stone score.^8^ Data was recorded in proformas by registrar urology including Perioperative data included variables such as puncture and tract entry site, mean operative time and perioperative complications and need for nephrostomy tube after the procedure, need for analgesics. Post-operative stone-free status was assessed on post-operative KUB radiograph and KUB ultrasound at three months post procedure. Complications were documented in line with the modified Clavien-Dindo classification system.^9^

### Procedure technique:

**Fig.1:**
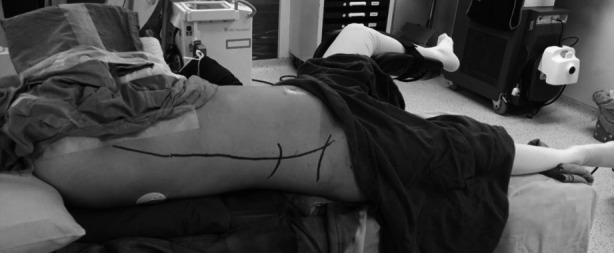
Supine PCNL in Giusti’s position.

All procedures were done under general anesthesia. The anatomical safety land marks were marked in neutral supine position i.e posterior axillary line, lowest rib and iliac crest (Fig.1). Patients were positioned in Cystoscopy position and ipsilateral ureteric orifice was catheterized and retrograde contrast study was done, to plan appropriate renal calyx access. Ureteric catheter was fixed with a Foleys catheter, placed in the urinary bladder. We used Giusti’s position,^6^ with the whole body slightly rotated 15 to 20 degrees towards the opposite side and this position was maintained by a folded cloth sheet, placed under the scapula and buttocks (Fig.1). Patients body was positioned about six centimeters from the edge of the table, to avoid table’s metal items interfering with C-arm fluoroscopy. Ipsilateral arm was rotated over the chest, to the opposite side.

**Fig.2:**
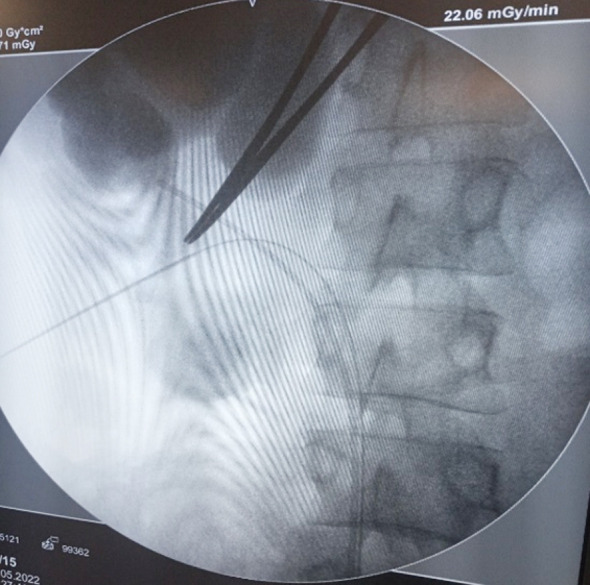
Supine PCNL via lower calyx puncture and hydrophilic guide wire is passed into the ureter.

The ipsilateral leg was kept in straight position while the opposite leg is placed in cystoscopy position. This position allows simultaneous retrograde endourology procedures, if required. We used fluoroscopy in all cases to access the appropriate calyx. Access needle was passed posterior to the posterior axillary line, nearly parallel to the floor (five patients required mid pole while 46 required lower pole puncture). Hydrophilic Guide wire 0.035 inches was passed, preferably in to the ureter and bladder (Fig.2). Tract was dilated using telescopic metallic dilators set and 26Fr or 30Fr Amplaz sheath was positioned. Pneumatic lithotripsy was done and fragments were removed with the grasping forceps. Supine position facilitated spontaneous passage of smaller stone fragments. Extra tract was needed in one patient only. Finally, antegrade JJ Stent was passed over the existing hydrophilic guide wire, under fluoroscopic control. No patient required Nephrostomy tube.

## RESULTS

Patients ages ranged from 21 to 69 years (mean 39 years), comprising of 62.74% male and 37.25% female patients. Thirty patients had their stones treated on the left side.Table-I. Most of the patients were slightly overweight. Table-I. Stone burden ranged from 2 to 7.3cms (mean 3.2 cm as shown in Table-I). Patients with congenital renal abnormalities were excluded from this study. Most of the stones were GUYs score one and two (complexity wise) as can be seen in Table-I. The procedure time ranged from 95 to 210 minutes (mean 147minutes, Table-II). Mean hospital stay of 2.17 days was observed in this study.

**Table-I:** Demographic variables.

Demographic Variables	Values
Number	51
Mean Age	39.17±13.49 years
Male	32 (62.74%)
Female	19 (37.25%)
Right Renal stone	21 (41.17%)
Left Renal stone	30 (58.82%)
Body Mass Index	27.16±5.35
Mean stone size (cm)	3.21±1.57 cm
Guys Stone Score	
Guys Stone Score 1	29 (%)
Guys Stone Score 2	18 (%)
Guys Stone Score 3	4 (%)
Guys Stone Score 4	0 (0%)

**Table-II:** Details of Procedure Outcomes.

Procedural outcomes	Values
Stone free rate	40 (78.44 %)
Residual stones	11 (21.56%)
Mean Operative time	147.37±61.29 minutes
Hospital stays	2.17±0.98 days
Nephrostomy	0/51
Double J stent	51/51

Only three patients (Table-III) required up to two units of packed RBCs if their Hemoglobin dropped below 9.0 gram/dl. One patient (2%) required Tranexamic acid post operatively, for persistent visible hematuria. Forty patients were stone free. Residual stones of more than 4mm size were seen in 11 (21.56%) patients, which could have been prevented by using Flexible Cystoscopy during the procedure, if available. Ancillary procedures including shock wave lithotripsy and flexible ureteroscopy laser stone procedures were needed in seven and four patients respectively. Only seven patients (14%) had level I-II complications (Clavien-Dindo classification). Mean follow up was three months (range 2-6 months). JJ Stent was passed in all patients and none had nephrostomy tube. JJ Stent was removed 4-6 weeks post PCNL, by flexible cystoscopy in the Clinic.

**Table-III:** Complications.

Complication grade	Type Complication	Number (%)
1	Fever	1 (1.4%)
1	Illeus without need NG tube	2/70(1.42%)
1	Transient hematuria	1/70 (11.4%)
2	Transfusion	3/70 (5.71%)
2	Sepsis	0 (0%)
3	Bowel injury	0%
3	Renal vascular injury requiring angioembolisation	0%
4	Septic Shock ICU manage	0 %
5	Death	0 %

## DISCUSSION

Supine PCNL was started in our hospital when it was required in a disproportionate dwarf patient with compromised pulmonary functions, who would drop his oxygen saturation on prone positioning. Supine PCNL contributes to about 20% of all PCNL worldwide.^10^ Supine PCNL has a short learning curve for an experienced endourologist.^10^,^11^ Both prone and supine PCNL have comparable total operating time, complications and stone free rate.^10^-^12^ Surgeon can sit comfortably during PCNL in supine position. Supine PCNL also reduces radiation exposure to Surgeon’s hand, compared to PCNL in prone position. Supine PCNL has low risk of injury to the colon.^13^ Supine PCNL generates low intrarenal pressure, easy gravity retrieval of stone fragments with lesser risk of infection.^12^ It is a very safe and convenient position from anesthesia point of view, with less fluid absorption in patients with compromised cardiovascular status.^13^ It improves ventilation in obese overweight patients.^14^ Unlike prone PCNL, it also provides a very convenient access to simultaneous performance of other transurethral endourology procedures, like retrograde intra renal surgery (RIRS) etc.^15^ However, a big drawback in supine PCNL is increased mobility of the kidney, especially in low body weight patients, in whom kidney is not fixed by the patients weight, as in prone PCNL. Supine PCNL may requires longer percutaneous tract, especially in obese patients requiring longer length Amplaz sheath and rigid Nephroscope.^16^

In a study by Ferreira et al. it was noted that during supine PCNL, only one thin (nonobese) patient (0.2%) encountered a colon perforation which was diagnosed intraoperatively and was subjected to conservative management by utilizing separate drainage of the urinary and gastrointestinal tracts with sufficiently good outcomes. Apart from this, they also encountered trans splenic puncture (0.2%) in one patient, which they satisfactorily managed by keeping patient on bed rest and a nephrostomy tube for seven days to help tamponade the punctured site. Pelvicalyceal injury was seen in their patients in 5.2% cases.^17^ They reported two deaths because of sepsis. In our study, only three patients (Table-III) required up to two units of packed RBCs as their Hemoglobin dropped below 9.0 gram/dl. One patient (2%) required Tranexamic acid post operatively, for persistent visible hematuria. Complications such as bowel injury or septic shock were not seen in our patients. In contrast to Ferreira et al, we did not encounter any death in our operated patients. This lesser rate of complication may be ascribed to the lower number of patients in our study as compared to them and probably we carefully selected patients. We can see in Table-I that 47/51 patients in our study had Guys Stone Score 1-2. This careful and less complex cases of stones in our study may be the factor for lower rate of complications.

In a study by Jamil MN et al. they attained stone clearance rate of 87% in supine position and 89% in the prone positioning group.^18^ In our study we achieved stone free rate of 40/51 (78.43%). They did not categorize stone complexity as we did according to Guys Stone Score but we may safely assume that probably we had initial experience of supine PCNL in this study and may be in future our results become much better. They observed transfusion in 3.56% while in our study 3/70 (5.71%) patients received transfusion. It is comparable to their results keeping in view our initial experience. In a study done by Choudhury S et al. the mean post operative hospital stay was 4.1 days while in present series it was 2.17±0.98 days. Table-II^19^

### Limitations and Strengths:

It was a retrospective single center study with a smaller sample size. More studies are required in future pertaining to the initial outcomes and learning difficulties in undertaking supine position PCNL at new centers. There is no study in Pakistan to best of our knowledge which addresses the learning curve of supine PCNL in a newly established hospital. This paper will help other centers regarding how the initial outcomes can be and how to proceed safely to progress the learning skills smoothly. No study in Pakistan has explained outcomes in terms of standard graded complexity of stones which we used to clearly explain complexity of stone location wise in kidney, which has an impact on outcomes of Supine PCNL. We have graded complications in standard Clavien-Dindo classification of surgical complications. In addition to treatment success, the numbers and severity of complications can be used as good indicators of competence.

## CONCLUSION

In our early preliminary experience, supine PCNL is an effective and safe treatment option, with results comparable with prone PCNL.

### Authors’ Contribution:

**NI, TA** and **EN:** Conceived, designed, and did statistical analysis & editing of the manuscript, is responsible for the integrity of research.

**SN, TA, EN** and **NI:** Did data collection and manuscript writing.

**NI, TA, SN** and **EN:** Did review and final approval of the manuscript.

**NI:** Responsible for the accuracy or integrity of the work.
